# Complementary Breath Profiling Using eNose and Gas Chromatography‐Mass Spectrometry for Noninvasive Triage of Indeterminate Pulmonary Nodules

**DOI:** 10.1002/mco2.71012

**Published:** 2026-09-23

**Authors:** Waqar Ahmed Afridi, Gunter Hartel, Xi Zhang, David Pass, David Fielding, Chamindie Punyadeera

**Affiliations:** ^1^ Saliva and Liquid Biopsy Translational Laboratory Institute for Biomedicine and Glycomics (IBG) Griffith University Brisbane Australia; ^2^ Virtual University of Pakistan Islamabad Pakistan; ^3^ QIMR Berghofer Medical Research Institute Brisbane Australia; ^4^ Public and Environmental Health Reference Laboratories (PEHRL) Brisbane Australia; ^5^ The Royal Brisbane and Women's Hospital Brisbane Australia

**Keywords:** biomarkers, eNose, gas chromatography‐mass spectrometry, lung cancer, pulmonary nodules, volatile organic compounds

## Abstract

Breath analysis may improve the noninvasive triage of CT‐detected pulmonary nodules. We prospectively evaluated two complementary breath platforms, an electronic nose (eNose) and gas chromatography‐mass spectrometry (GC‐MS), in patients with newly detected nodules. The eNose cohort included 89 participants (55 malignant and 34 benign), and the GC‐MS cohort included 75 participants (52 malignant and 23 benign). For each platform, three logistic regression models were assessed: clinical‐only, biomarker‐only, and combined clinical + biomarker. For eNose, apparent discrimination improved from the clinical‐only model (AUC 0.67; misclassification rate [MCR] 40.4%) to the sensor‐only model (AUC 0.86; MCR 22.5%), with further improvement in the combined clinical + sensor model (AUC 0.88; MCR 22.5%). Internal validation showed attenuation, with the combined eNose model achieving a leave‐one‐out cross‐validation (LOOCV) AUC of 0.67. For GC‐MS, LASSO logistic regression showed strong apparent discrimination for the VOC‐only (AUC 0.97; MCR 4%), although internal validation indicated attenuation of performance (bootstrap‐corrected AUC 0.64; LOOCV AUC 0.65). The combined clinical + VOC model remained comparably high (AUC 0.96; MCR 8%), outperforming the clinical‐only benchmark (AUC 0.73; MCR 32%). These findings support complementary, noninvasive breath‐based approaches for pulmonary nodule triage and warrant larger multicenter validation studies.

## Graphical abstract

Flow diagram illustrating participant inclusion and exclusion for the eNose and GC‐MS analyses. Of the 98 prospectively enrolled participants, 79 were eligible for the eNose analysis after the exclusion of 14 participants because of procedural or eligibility issues, four without breath samples, and one without a valid eNose recording. Ten historical benign controls were subsequently added, yielding a final eNose cohort of 89 participants (55 malignant and 34 benign). For the GC‐MS analysis, 75 of the 98 prospectively enrolled participants were included after the exclusion of 14 participants because of procedural or eligibility issues, four without breath samples, and five without valid GC‐MS data, resulting in a final cohort of 52 malignant and 23 benign participants.

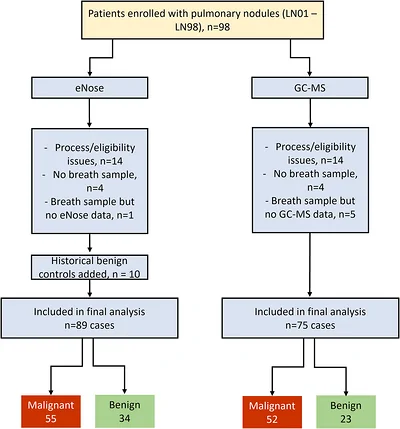

## 1 Introduction

Lung cancer remains a major global health challenge, with approximately 2.5 million new cases diagnosed annually [1, 2, 3]. Annual low‐dose CT screening is advised for high‐risk individuals as a proactive measure to combat this escalating issue. In the United States alone, millions of chest CT scans are conducted each year [4]. However, up to 95% of pulmonary nodules identified on low‐dose CT scans, whether found during screening or clinical evaluation, are false positives. In other words, these are lesions that initially appear suspicious but are ultimately determined to be non‐cancerous [5]. These findings complicate clinical decision‐making, often leading to unnecessary biopsies and, in some cases, avoidable surgeries [6, 7]. Biopsying malignant nodules presents additional challenges. For example, when lesions are often small and technically difficult to access, the procedure carries risks such as pneumothorax, particularly in patients with emphysema [8]. In contrast, delaying biopsy can negatively impact outcomes, as early detection significantly improves survival, which is approximately 70% for localized disease versus 15% for advanced‐stage cancer [9, 10].

Current imaging modalities are unable to accurately distinguish benign from malignant nodules. As a result, researchers are actively pursuing novel biomarkers to improve triage and reduce reliance on invasive procedures [11, 12]. Any biomarker‐based approach must demonstrate high sensitivity and specificity, be cost‐effective, and be seamlessly integrated into routine clinical workflows. Ideally, such biomarkers should deliver timely, actionable results that minimize the need for procedures such as CT‐guided biopsy, bronchoscopy, or surgery [13, 14].

Breath testing for volatile organic compounds (VOCs) is gaining momentum, as these metabolic by‑products exhibit profile changes in response to various disease states, including cancer [15, 16]. Many VOCs detected in lung cancer directly reflect underlying biological processes, such as lipid peroxidation, oxidative stress, and exposure‐related xenobiotics, providing a mechanistic basis for their diagnostic relevance [17]. Breath samples may be analyzed using portable breath devices or advanced laboratory methods, such as gas chromatography‐mass spectrometry (GC‐MS), to identify VOC signatures associated with malignant nodules [18, 19]. Electronic nose (eNose) technology is becoming increasingly popular because of its ease of use and its ability to leverage pattern‑recognition algorithms to identify disease‑specific “smellprint signatures” [20, 21]. Beyond lung cancer, semiconductor‐based eNose platforms have also demonstrated promising diagnostic performance for respiratory diseases such as SARS‐CoV‐2 pneumonia, further highlighting the broader clinical potential of breath‐based point‐of‐care diagnostics [22]. In pulmonary nodule workflows, the eNose can noninvasively aid in the triage of CT‐detected nodules by prioritizing those with a higher malignancy risk for further investigation and reducing the urgency for those likely to be benign when used alongside standard clinical risk models. Recent prospective and multicenter validation studies have reported high diagnostic accuracy and real‐world feasibility of the eNose in a thoracic clinic setting [23].

Despite the development of multiple breath‑analysis platforms, including ion mobility spectrometry, selected ion flow tube mass spectrometry, and laser spectrometry, many of these technologies remain too complex for routine clinical use [24]. We previously demonstrated that VOCs could detect small lung cancers confirmed via bronchoscopy or laryngoscopy, including radiologically occult carcinoma in situ, with 95% sensitivity and 65% specificity [25]. VOC alterations distinguished in situ cancers from both controls and advanced cancers, and eNose‐based models achieved strong performance (sensitivity 95%, specificity 69%). Concurrent GC‐MS analysis identified several VOCs unique to the in situ cohort. Bajo‐Fernández et al. [26] used breath samples from biopsy‐confirmed lung cancer patients and reported substantial variability in sampling, pre‐concentration, and analytical workflows. Numerous groups have attempted to define VOC signatures for lung cancer detection [27, 28, 29, 30, 31]. However, these VOCs have rarely been replicated in independent cohorts, which limits their clinical translation [32]. Differences in breath‐collection devices, sorbents, chromatographic columns, spectral libraries, and statistical methods have led to poor cross‐study agreement [33]. Many reported markers may reflect methodological artifacts or environmental contamination rather than true disease‐associated VOCs [34, 35]. External validation is rare, cohorts are often small or heterogeneous, and few findings are independently replicated.

To overcome these challenges, we developed a standardized GC‐MS workflow that integrates defined contaminant removal and quality control procedures to improve reproducibility and facilitate future multicenter breath studies. Building on our prior research, we conducted an independent pilot study of breath VOC testing and eNose analysis in patients undergoing evaluation for indeterminate pulmonary nodules [25]. Breath samples were collected before biopsy, and we evaluated whether clinical variables (including the Brock score) could be combined separately with eNose‐ and GC‐MS‐derived predictors to estimate nodule risk. We intentionally did not construct an integrated eNose + GC‐MS meta‐model in this study, as the primary objective was to establish and internally evaluate the performance of each platform individually within the same clinical cohort. These findings are intended to inform the development of future staged or integrated assays once larger multicenter datasets become available.

## 2 Results

### 2.1 Breath eNose

A total of 98 participants with CT‐detected pulmonary nodules (LN01‐LN98) were enrolled. After the quality‐control filters and data‐availability criteria were applied, 89 participants were included in the eNose analyses, and 75 were included in the GC‐MS analyses (Figure S1).

#### 2.1.1 Cohort Characteristics

The eNose analysis set included 89 participants. Malignant cases exhibited higher Brock scores (30.4 vs. 21.4; *p* = 0.0073) and slightly larger nodules (17.1 mm vs. 14.4 mm; *p* = 0.0425), whereas age, sex, and Eastern Cooperative Oncology Group (ECOG) performance status were comparable between the groups (Table 1).

**TABLE 1:** Metadata and clinicopathological characteristics of the study cohort used for eNose analysis.

Category	Characteristics	Benign (*n* = 34)	Malignant (*n* = 55)	*p*‐value
Demographics	Age, mean (SD), years	68.3 (8.8)	69.6 (8.5)	0.506^a^
	Sex (male), *n* (%)	21 (61.8%)	28 (50.9%)	0.315^b^
Smoking history	Current smoker, *n* (%)	11 (32.4%)	24 (43.6%)	0.287^b^
	Ex‐smoker, *n* (%)	23 (67.6%)	31 (56.4%)	—
Clinical features	Nodule diameter, mean (SD), mm	14.4 (6.3)	17.1 (4.9)	0.042^a^
	Brock score, mean (SD)	21.4 (11.0)	30.4 (14.2)	0.007^a^
	ECOG 0, *n* (%)	21 (61.8%)	38 (69.1%)	0.143^b^
	ECOG 1, *n* (%)	9 (26.5%)	16 (29.1%)	—
	ECOG 2, *n* (%)	4 (11.8%)	1 (1.8%)	—
Histopathological/clinical diagnosis^c^	Adenocarcinoma *n* (%)	—	26 (47.3%)	—
	NSCLC‐NOS *n* (%)	—	12 (21.8%)	—
	Squamous‐cell carcinoma *n* (%)	—	8 (14.5%)	—
	Small‐cell carcinoma *n* (%)	—	2 (3.6%)	—
	Metastatic from other organ *n* (%)	—	1 (1.8%)	—
	Lymphoma *n* (%)	—	1 (1.8%)	—
	Other malignant *n* (%)	—	5 (9.0%)	—
	Radiologically stable/shrinking *n* (%)	9 (36.0%)	—	—
	Granulomatous infection *n* (%)	5 (20.0%)	—	—
	Acute bacterial infection *n* (%)	4 (16.0%)	—	—
	Carcinoid (benign) *n* (%)	3 (12.0%)	—	—
	Other benign *n* (%)	4 (16.0%)	—	—
Totals for the diagnosis section		25 (100%)	55 (100%)	—

*Note*: Values are presented as mean (SD) or *n* (%).

^a^ Two‐sample t‐test.

^b^ Likelihood‐ratio *χ* ^2^ test.

^c^ The eNose analysis cohort comprised 89 participants (55 malignant and 34 benign), including 24 prospectively recruited benign participants with usable eNose data and 10 historical benign controls. Histopathological and clinical diagnostic categories are reported for the complete prospectively recruited cohort (*n* = 80; 55 malignant and 25 benign), including one benign participant without a usable eNose recording who was retained in the GC‐MS analysis. GC‐MS data were available for 75 participants after modality‐specific exclusions.

Histopathological and clinical diagnoses for the prospectively collected cohort (*n* = 80) were predominantly adenocarcinoma (26/55, 47.3%), followed by non‐small cell lung cancer not otherwise specified (NSCLC‐NOS) (12/55, 21.8%) and squamous‐cell carcinoma (8/55, 14.5%), with smaller numbers of small‐cell carcinoma (2/55, 3.6%), metastatic malignancy (1/55, 1.8%), lymphoma (1/55, 1.8%), and other malignant types (5/55, 9.0%). Among benign nodules (*n* = 25), radiologically stable or shrinking lesions (9/25, 36.0%) were most common, followed by granulomatous infections (5/25, 20%), acute bacterial infections (4/25, 16%), carcinoids (3/25, 12%), and other benign etiologies (4/25, 16%). Although current smoking status was more common among malignant patients than among benign patients (43.6% vs. 32.4%), the difference was not statistically significant (*p* = 0.287; Table 1).

Breath data were available from both modalities within the same participant cohort. Among the 98 enrollees, 89 had usable eNose data; one participant (LN07) lacked a valid eNose recording but contributed to the GC‐MS analyses; and 75 had valid GC‐MS spectra. Four participants (LN36, LN38, LN66, and LN81) had no breath samples, and five (LN43, LN44, LN46, LN84, and LN91) produced eNose data but no GC‐MS output and were therefore excluded from GC‐MS analyses, yielding the final 75‐case GC‐MS dataset. In addition, 14 participants (LN04, LN11, LN14, LN19, LN20, LN22, LN30, LN31, LN32, LN35, LN48, LN53, LN57, and LN59) were excluded at earlier stages because of procedural or eligibility issues. These sample counts and modality‐specific exclusions are summarized in Figure S1, whereas demographic comparisons are presented in Table 1. Details of GC‐MS compound selection and model performance are presented in Section 2.2.

#### 2.1.2 Model Performance

This section summarizes the discriminatory performance of the eNose‐based logistic regression models. The performance of the clinical‐only and eNose sensor‐only models is described in the main manuscript, with the corresponding receiver operating characteristic (ROC) curves shown in Figures S2 and S3, respectively. The main manuscript presents the results for the combined clinical + eNose model, together with the overall comparative performance across the three models. Pairwise areas under the curve (AUC) comparisons between the eNose models are reported in Table S1.

##### 2.1.2.1 Predictive Value of Clinical Parameters Alone (Clinical‐Only Model)

The clinical‐only model (age, sex, smoking history, and ECOG) achieved an AUC of 0.67 (95% CI [confidence interval] 0.547–0.773) across the full cohort (*n* = 89), with the ROC curve shown in Figure S2. This represents modest discrimination between benign and malignant nodules. Because the Brock score and nodule diameter were unavailable for the 10 historical benign controls, a sensitivity analysis including these parameters but restricted to the prospectively collected subset (*n* = 79) increased the AUC to 0.73, although overall discrimination remained limited (Table 2).

**TABLE 2:** Discrimination performance of the clinical‐only, eNose sensor‐only, and combined (clinical + eNose sensor) models.

Model	AUC (95% CI)	Misclassification rate
Clinical‐only	0.67 (0.547–0.773)	40.4%
eNose sensor‐only	0.86 (0.755–0.920)	22.5%
Combined (clinical + eNose sensor)	0.88 (0.788–0.933)	22.5%

*Note*: Model metrics derived from adaptive LASSO logistic regression.

##### 2.1.2.2 Predictive Value of eNose Sensors Alone (eNose Sensor‐Only Model)

The eNose sensor‑only model selected 10 of the 32 sensors using adaptive least absolute shrinkage and selection operator (LASSO) logistic regression and showed markedly superior diagnostic performance compared with the clinical model. The eNose model achieved an AUC of 0.86 (95% CI 0.755–0.920) in the full cohort (*n* = 89), with a misclassification rate (MCR) of 22.5%, indicating good discrimination. The ROC curve for the eNose sensor‐only model is shown in Figure S3, and the corresponding performance metrics are provided in Table 2.

##### 2.1.2.3 Predictive Value of the Combined Model (Clinical + eNose Sensor)

**FIGURE 1:**
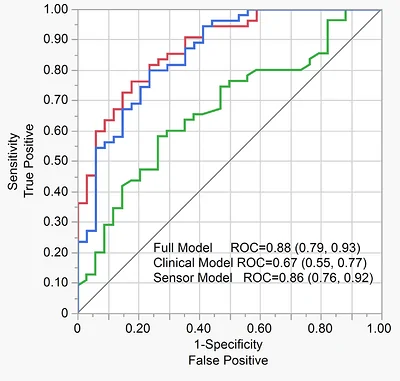
Receiver operating characteristic (ROC) curves for the eNose models. The clinical‐only model demonstrated modest discrimination (AUC 0.67; 95% CI 0.55–0.77). The eNose sensor‐only model showed substantially stronger apparent discrimination (AUC 0.86; 95% CI 0.76–0.92). The combined clinical + eNose model achieved the highest apparent performance (AUC 0.88; 95% CI 0.79–0.93). All curves are shown on the same axes to enable direct comparison of model performance.

The combined clinical + eNose model showed high apparent discrimination (AUC 0.88), with an MCR of 22.5%. The ROC curve for the combined model is presented in Figure 1, and the corresponding summary statistics are provided in Table 2.

##### 2.1.2.4 Comparative Performance Across Models

Discriminatory performance increased progressively across the three eNose models. The clinical‐only model demonstrated modest discrimination (AUC 0.67), whereas the eNose sensor‐only model showed substantially stronger apparent discrimination (AUC 0.86). The combined clinical + eNose model achieved the highest apparent performance (AUC 0.88). Although the eNose sensor‐only and combined models performed similarly in terms of apparent AUC, internal validation indicated attenuation of performance, with the combined model retaining a measurable discriminatory signal under leave‐one‐out cross‐validation (LOOCV). These relationships are summarized in Figure 1, with corresponding numerical summaries provided in Table 2. Formal AUC comparisons between the three eNose models are reported in Table S1.

#### 2.1.3 Stability and Importance of Predictors

**FIGURE 2:**
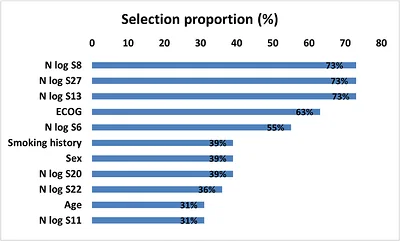
Variable importance of eNose predictors across 1000 bootstrap resamples. The bars show the inclusion proportion (%) from adaptive LASSO models. Only predictors meeting the stability threshold (≥ 30%) are displayed, representing repeatedly selected predictors distinguishing malignant from benign pulmonary nodules.

Using a ≥ 30% stability threshold, three sensors (S8, S27, S13; each 73%) were the most consistently selected predictors, followed by ECOG (63%) and S6 (55%). Additional retained predictors showed moderate stability: smoking history (39%), sex (39%), S20 (39%), S22 (36%), age (31%), and S11 (31%). Collectively, these findings indicate that a subset of eNose sensor patterns, complemented by key clinical covariates, was repeatedly selected across resamples and retained discriminatory relevance for distinguishing malignant from benign pulmonary nodules. The inclusion proportions for these predictors are visualized in Figure 2, with the full stability frequencies reported in Table S2.

#### 2.1.4 Internal Validation

Efron's 0.632+ bootstrap validation indicated moderate optimism (*R* = 0.60), yielding an optimism‐corrected AUC of 0.69 (95% CI 0.58–0.80). Under outer‐loop LOOCV, the combined model achieved an AUC of 0.67 (95% CI 0.561–0.784), as shown in Figure S4.

Overall, these findings indicate that integrating breath‐based sensor signals with clinical predictors retained measurable discriminatory information under internal validation, while also highlighting the need for further optimization and external validation in larger, independent cohorts.

### 2.2 GC‐MS Results

#### 2.2.1 Feature Extraction and Compound Selection

As detailed in Section 5, the GC‐MS chromatograms were processed and aligned to generate the working feature set. At acquisition, approximately 1500 candidate VOC signals were detected per run; after cross‐sample consolidation across cohorts, 296 unique VOC features were retained for evaluation. Applying the presence filter (detected in ≥ 30% of participants) yielded 48 VOC features suitable for modeling. These retained features spanned carbonyls, alkanes/alkenes, aromatics, and halogenated or sulfur‐containing compounds, several of which were repeatedly selected across LOOCV runs, supporting panel‐level stability and biological coherence (Section 2.2.4).

#### 2.2.2 Compound Identification and Statistical Filtering

Compound annotation, prevalence filtering (≥ 30%), and binary feature encoding were performed as detailed in Section 5. Eleven VOC features were nominally significant (*p* < 0.05) in discriminating benign from malignant pulmonary nodules based on empirical‐Bayes group comparisons. These compounds included carbonyls (e.g., cyclohexanone, C_6_H_10_O), oxygenated heterocycles (e.g., 3‐methylfuran, C_5_H_6_O), alkanes and alkenes (C_5_H_12_, C_6_H_14_, C_6_H_12_, C_7_H_14_, C_8_H_18_), an aromatic hydrocarbon (C_8_H_10_), and exogenous solvents (C_2_Cl_4_, C_2_HCl_3_, CS_2_). All eleven features were detected more frequently in malignant samples than in benign samples.

The full list of discriminative VOCs (nominal *p* < 0.05), together with *p*‐values and detection directions, is presented in Table 3. This table summarizes only the hypothesis‐testing results. Subsequent assessments of model stability and variable importance, based on 1000 bootstrap resamples and reported as inclusion proportions, are provided in Section 2.2.5 and Figure 5. Several of the VOCs listed in Table 3 also exhibited high inclusion frequencies in those analyses, supporting their relevance as candidate biomarkers.

**TABLE 3:** Discriminative GC‐MS VOC features (nominal *p* < 0.05): Molecular formulas, putative compound examples, chemical classes, *p*‐values, and direction of change. The arrows indicate a higher detection frequency in malignant cases.

Formula	Putative compound/example	Chemical class	*p*‐value	Direction (malignant vs. benign)
C_6_H_10_O	Cyclohexanone	Oxygenated compound	0.002	↑ Malignant
C_5_H_6_O	3‐methylfuran	Oxygenated heterocycle	0.004	↑ Malignant
C_5_H_12_	Pentane isomer	Alkane	0.006	↑ Malignant
C_6_H_14_	Hexane isomer	Alkane	0.008	↑ Malignant
C_6_H_12_	Hexene/cycloalkane isomer	Alkene	0.014	↑ Malignant
C_7_H_14_	Heptene/cycloalkane isomer	Alkene	0.013	↑ Malignant
C_2_Cl_4_	Tetrachloroethylene	Chlorinated solvent	0.031	↑ Malignant
C_2_HCl_3_	Trichloroethylene	Chlorinated solvent	0.037	↑ Malignant
CS_2_	Carbon disulfide	Sulfur‐containing compound	0.042	↑ Malignant
C_8_H_10_	Ethylbenzene/xylene isomer	Aromatic hydrocarbon	0.043	↑ Malignant
C_8_H_18_	Octane isomer	Alkane	0.048	↑ Malignant

*Note*: “↑” indicates a higher detection frequency in malignant cases. Features are reported at the molecular formula level using binary present/absent encoding because of the semi‐quantitative nature of the GC‐MS assay. Although this approach simplifies feature harmonization across samples, it may reduce the amount of quantitative information and limit transportability, requiring validation in larger independent cohorts. Putative compound examples are based on spectral library matching and molecular formulas and should be interpreted with caution. *p*‐values are derived from empirical‐Bayes group comparisons (nominal, unadjusted for multiple testing) and are not model coefficients. Overall, this pattern indicates a higher detection frequency of these features in malignant cases, including compounds potentially associated with oxidative stress, lipid peroxidation, membrane damage, and exposure‐related xenobiotic pathways.

#### 2.2.3 Model Performance

**FIGURE 3:**
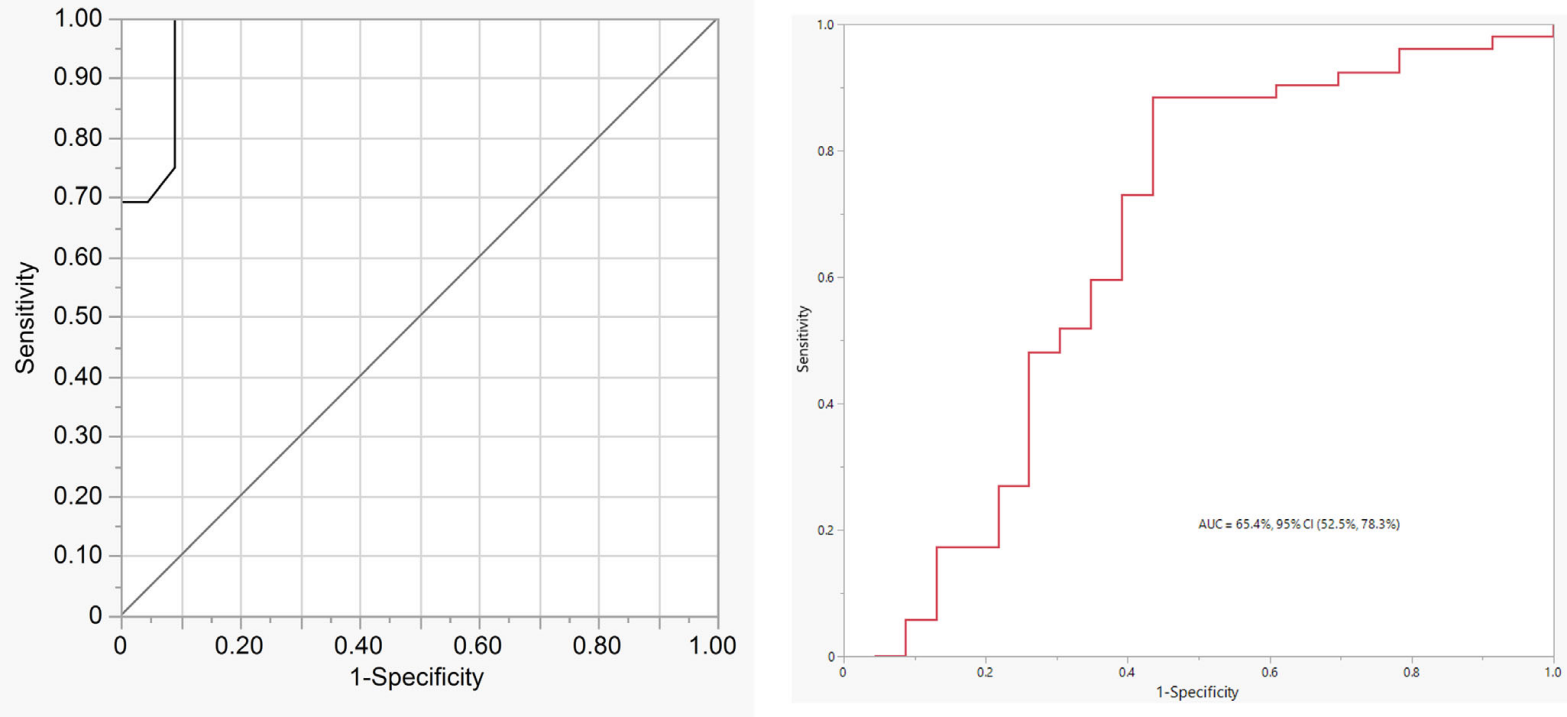
ROC curves for the GC‐MS VOC‐only model. (A) Training‐set ROC curve showing strong apparent discrimination between malignant and benign pulmonary nodules (AUC 0.97; 95% CI 0.90–0.99). (B) ROC curve derived from outer‐loop leave‐one‐out cross‐validation (LOOCV), representing internally validated predictive performance (AUC 0.65; 95% CI 0.53–0.78).

**FIGURE 4:**
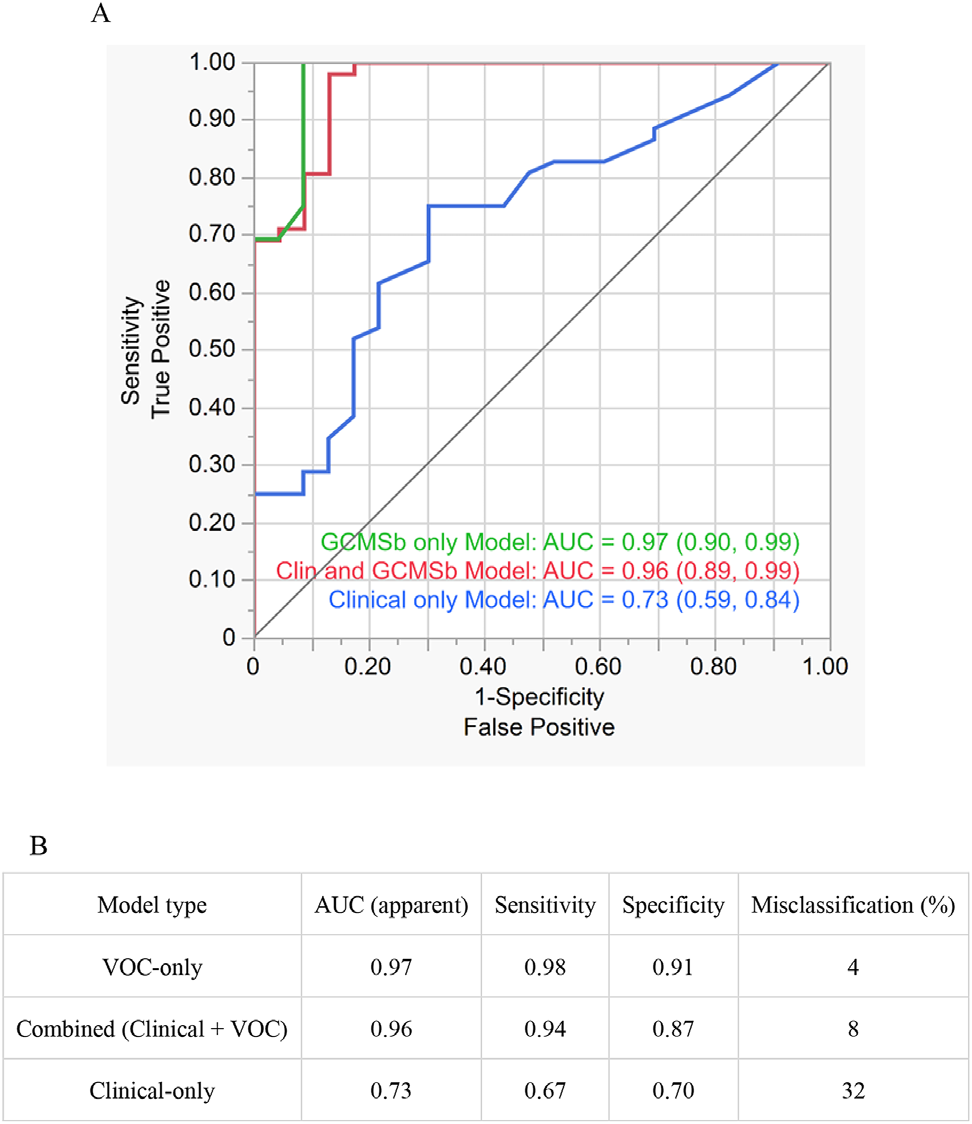
(A) Receiver operating characteristic (ROC) curves for the three GC‐MS models, showing the clinical‐only model (AUC 0.73), the VOC‐only model (AUC 0.97), and the combined clinical + VOC model (AUC 0.96). The overlaid ROC curves illustrate the substantial improvement in apparent discrimination following the inclusion of VOC data, with similar apparent performance observed for the VOC‐only and combined clinical + VOC models. (B) Performance metrics for the clinical‐only, VOC‐only, and combined clinical + VOC models, including apparent AUC, sensitivity, specificity, and misclassification rate.

To evaluate discriminatory performance, three logistic‐regression models were compared: (i) the clinical‐only model, (ii) the VOC‐only model, and (iii) the combined clinical + VOC model. The ROC curves for the VOC‐only and combined models are shown in the main manuscript (Figures 3 and 4), whereas the ROC curve for the clinical‐only model is provided in Figure S5. Pairwise AUC comparisons for the GC‐MS models are reported in Table S3.

##### 2.2.3.1 Predictive Value of Clinical Parameters Alone (Clinical‐Only Model)

The clinical‐only logistic regression model showed modest discrimination (AUC = 0.73), with an MCR of 32% at the decision threshold of 0.637. The corresponding sensitivity and specificity were 0.67 and 0.70, respectively. The ROC curve for the GC‐MS clinical‐only model is shown in Figure S5. The comparative performance of all three GC‐MS models is presented in Figure 4A, with the corresponding performance metrics summarized in Figure 4B.

##### 2.2.3.2 Predictive Value of VOCs Alone (VOC‐Only Model)

The VOC‐only logistic regression model constructed from GC‐MS VOCs demonstrated high apparent discrimination between malignant and benign pulmonary nodules (AUC 0.97, MCR 4%, sensitivity 0.98, and specificity 0.91), as shown by the training‐set ROC curve (Figure 3A). However, Efron's 0.632+ bootstrap validation indicated substantial optimism, yielding an optimism‐corrected AUC of 0.64 (95% CI 0.50–0.78). Under outer‐loop LOOCV, the predictive performance was attenuated (AUC 0.65; 95% CI 0.53–0.78), as shown in Figure 3B.

##### 2.2.3.3 Predictive Value of the Combined Model (Clinical + VOC)

The combined clinical + VOC model showed high apparent discrimination (AUC 0.96), with an MCR of 8%, sensitivity of 0.94, and specificity of 0.87. As with the VOC‐only model, apparent performance exceeded the internally validated performance. Bootstrap validation yielded an optimism‐corrected AUC of 0.66 (95% CI 0.53–0.80). The ROC curve for this model is presented in Figure 4A.

##### 2.2.3.4 Comparative Performance Across Models

Discriminatory performance improved substantially following the inclusion of VOC predictors. The clinical‐only model demonstrated modest discrimination (AUC 0.73), whereas both the VOC‐only model (AUC 0.97) and the combined clinical + VOC model (AUC 0.96) showed high apparent discrimination. Although the VOC‐only model yielded a marginally higher apparent AUC, incorporating clinical variables improved the internally validated performance and partially mitigated the attenuation observed in the VOC‐only model. These comparative relationships are illustrated in Figure 4A, with the corresponding performance metrics summarized in Figure 4B.

#### 2.2.4 Functional Analysis of Key VOC Classes

Frequently detected VOCs, as prioritized by the GC‐MS models, corresponded to metabolic pathways with established relevance to lung cancer. Many compounds fall into broadly recognized chemical categories such as carbonyls, oxygenated heterocycles, alkanes/alkenes, and chlorinated solvents, which are consistent with patterns commonly reported in breath VOC studies. A summary of these VOCs, their putative chemical classes, and reported biological associations is provided in Table S4.

#### 2.2.5 Stability and Importance of Predictors

**FIGURE 5:**
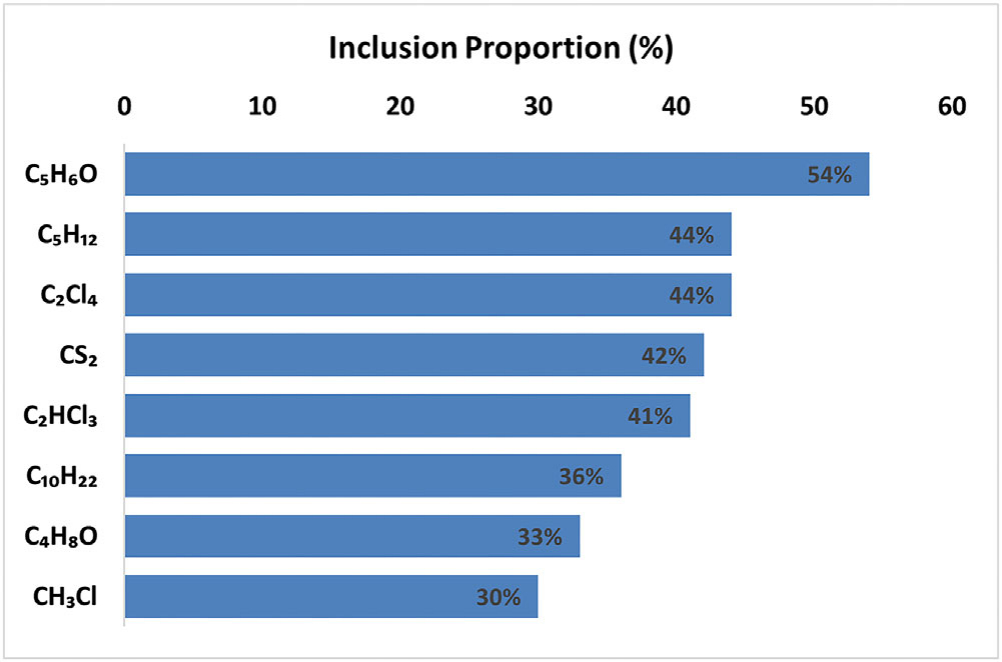
Variable importance of GC‐MS‐derived VOC predictors across 1000 bootstrap resamples. The bars show the inclusion proportion (%) from adaptive LASSO models. Only compounds meeting the stability threshold (≥ 30%) are displayed, representing stable and reproducible predictors distinguishing malignant from benign pulmonary nodules.

Stability analysis revealed a compact subset of eight VOCs that were consistently retained above the predefined ≥ 30% inclusion threshold: C_5_H_6_O (54%), C_5_H_12_ (44%), C_2_Cl_4_ (44%), CS_2_ (42%), C_2_HCl_3_ (41%), C_10_H_22_ (36%), C_4_H_8_O (33%), and CH_3_Cl (30%). These predictors were repeatedly selected across resamplings, indicating a stable contribution to the GC‐MS model. The corresponding selection frequencies are shown in Figure 5.

## 3 Discussion

Breath analysis is an emerging method for differentiating pulmonary nodules and has the potential to support minimally invasive triage. In this study, eNose sensor signatures and GC‑MS‐derived VOC profiles were evaluated concurrently in the same clinically relevant cohort, without developing an integrated eNose‐GC‑MS meta‑model. The sensor‐only model showed strong apparent discrimination (AUC 0.86), which improved to 0.88 when combined with clinical data; however, internal validation indicated attenuation, with the combined model achieving an LOOCV AUC of 0.67. For GC‐MS, the VOC‐only model showed high apparent discrimination (AUC 0.97), but internal validation revealed substantial attenuation (bootstrap‐corrected AUC 0.64; LOOCV AUC 0.65), consistent with the optimism expected in high‐dimensional pilot biomarker studies. Notably, adding clinical variables modestly improved the optimism‐corrected performance of the GC‐MS model (AUC 0.66), suggesting that clinically grounded multimodal modeling may partially recover performance loss after validation. Importantly, incorporating clinical variables improved model stability and partially mitigated the performance loss observed in VOC‐only models.

A key finding of this study is the marked difference between apparent model performance and internally validated performance. For the GC‐MS model, the apparent AUC of 0.97 decreased to approximately 0.64 following bootstrap correction, whereas for the eNose model, the apparent AUC of 0.88 decreased to approximately 0.67 under LOOCV. This degree of optimism is not unexpected in biomarker studies characterized by modest sample sizes and a relatively large pool of candidate predictors. Accordingly, these findings should be viewed as proof‐of‐concept rather than evidence of clinical readiness and require validation in larger, independent cohorts. Notably, the enhanced performance of clinically augmented models suggests that breath‐derived molecular signals may offer greater diagnostic value when interpreted alongside established clinical variables.

The combined clinical + VOC model maintained high apparent performance (AUC 0.96), but this finding should be interpreted alongside the optimism‐corrected performance estimate. Predictor stability was defined as inclusion in ≥ 30% of 1000 adaptive LASSO bootstrap refits. Together, these findings support the evaluation of complementary breath‐based approaches for pulmonary nodule triage and highlight the importance of cautious interpretation following internal validation. Our findings align with prior research on the diagnostic potential of breath VOCs in lung cancer [36, 37]. The major strengths of our study include: (i) prospective accrual within a clinically relevant pulmonary nodule cohort undergoing diagnostic work‐up; (ii) the application of two complementary breath analysis platforms, pattern recognition eNose and chemistry‐resolved GC‐MS, within the same participants; (iii) transparent, regularized modeling with predefined thresholds, ROC‐AUC, and internal validation using both LOOCV and Efron's 0.632+ bootstrap; and (iv) alignment with reporting principles derived from frameworks such as TRIPOD‐AI and STARD [38].

A recent systematic review and meta‐analysis incorporating 25 studies reported a pooled AUC of 0.90 for distinguishing benign from malignant nodules using VOCs [16]. Similarly, mass‐spectrometry‐based perioperative dynamic breathomics has demonstrated strong discriminatory performance and biologically coherent shifts in VOC profiles, underscoring the value of chemistry‐resolved readouts alongside sensor‐based pattern recognition [39]. Evidence from earlier eNose studies similarly highlights the importance of careful model development and validation when analyzing heterogeneous clinical datasets [40]. In the present study, the use of penalized regression together with bootstrap and cross‐validation procedures helped quantify optimism and define a more realistic estimate of predictive performance. Methodologically, our modeling strategy, penalized logistic regression with LOOCV and bootstrap optimism correction, aligns with previous VOC modeling studies in indeterminate nodules, where adding clinical covariates improved the calibration and stability, which is consistent with our combined models [21].

In a blinded validation cohort of 161 participants undergoing LDCT, Phillips et al. [31] evaluated exhaled VOCs using their single‐ion biomarker (“MAGIIC”) strategy. The marker achieved 84% accuracy for detecting biopsy‐proven lung cancer (sensitivity 75.4%, specificity 85.0%) and approximately 80% accuracy for predicting CT‐detected pulmonary nodules (sensitivity 80.1%, specificity 75.0%). Although their study highlighted the promise of breath‐based VOC profiling for early lung lesion detection, the absence of lesion size or tumor stage data limits comparison with our < 3 cm indeterminate nodule cohort. Nakhleh et al. [41] used a nanoparticle‐based sensor array in 1404 participants spanning 17 diseases and healthy controls (overall accuracy ≈ 86%), including 45 lung cancer cases. However, lesion size and tumor stage were not specified, precluding assessment of early‐stage performance. Collectively, these studies highlight both the diagnostic promise and methodological heterogeneity of previous breath‐VOC research, while underscoring the distinctiveness of our focus on indeterminate nodules in a real‐world clinical setting.

A GC‐MS breathomics review across cancer and lung disease established it as the benchmark platform, supporting the development and validation of our VOC panel [26]. Recent advances in metabolomics have outlined best practices for identifying high‐confidence breath VOCs against reference standards, with a strong emphasis on background control, principles that guided our data curation and feature selection [42]. Biologically, the VOCs retained in our GC‐MS models align with relevant metabolic pathways. Oxygenated compounds (e.g., cyclohexanone, 3‐methylfuran) indicate lipid peroxidation and oxidative stress, while alkanes and alkenes may reflect membrane damage and redox imbalance [43, 44]. Xenobiotic markers (e.g., tetrachloroethylene and carbon disulfide) may capture environmental or exposure‐related signals that contribute orthogonal discriminatory information [45, 46]. Stability analyses identified a compact, biologically coherent VOC panel (e.g., C_5_H_6_O, C_2_Cl_4_) as key drivers. Internal validation (bootstrap AUC = 0.64; LOOCV ≈ 0.65) indicated moderate but interpretable discrimination rather than robustness. Importantly, although the GC‐MS findings require cautious interpretation because of the high‐dimensional modeling context, the recurrent VOC classes were not biologically inert; several align with oxidative stress, lipid peroxidation, membrane damage, and xenobiotic‐exposure pathways previously reported in cancer and respiratory disease [43, 44, 45, 46]. The eNose models also showed consistent LOOCV performance, supporting cross‐platform reliability. Clinically, eNose may offer rapid point‐of‐care triage, whereas GC‐MS may provide additional molecular specificity for indeterminate nodules. Although these platforms were not combined in the present study, their parallel evaluation suggests a potential future staged workflow in which rapid eNose screening could be followed by GC‐MS molecular characterization. This possibility now requires external validation in larger multicenter cohorts before any clinical implementation can be considered. Our approach aligns with best practices in breathomics and meets the growing interest in VOC‐based lung cancer screening [47].

Our study also has several limitations, including (i) the single‐center design and modest sample size, which may introduce optimism despite resampling corrections; (ii) the dichotomization of GC‐MS features (present/absent), which simplifies noise but may reduce quantitative information and limit cross‐platform transportability, requiring validation in larger cohorts; (iii) the absence of external calibration, decision‐curve analysis, and cost‐utility analyses; and (iv) potential confounding by smoking history and comorbidities, which is consistent with systematic reviews showing that VOC shifts are linked to airway inflammation and COPD risk factors [48]. Finally, we intentionally refrained from constructing an integrated eNose + GC‐MS meta‐model, because our primary aim was to establish and internally evaluate the performance of each platform separately within the same clinical cohort. This conservative approach preserves interpretability and provides a clear foundation for future multicenter, integrated validation studies. In summary, our findings support the feasibility of breath‐based assays as complementary tools for triaging indeterminate pulmonary nodules and warrant validation in larger, prospective studies. These findings also highlight the importance of distinguishing between training‐set performance and internally validated performance when high‐dimensional biomarker models are interpreted.

## 4 Conclusion

Our study showed that eNose pattern recognition and GC‐MS VOC profiling provide complementary, noninvasive diagnostic information for differentiating benign from malignant pulmonary nodules. No integrated eNose + GC‐MS meta‐model was constructed in this study; instead, the two platforms were evaluated in parallel within the same clinical cohort. These findings provide proof‐of‐concept evidence supporting a future staged workflow in which rapid eNose screening could be followed by GC‐MS molecular characterization. However, internal validation demonstrated attenuation of model performance, indicating that these findings should be interpreted cautiously. Larger multicenter studies are needed to validate this approach, confirm its generalizability, and assess its clinical utility before its clinical implementation.

## 5 Methods

### 5.1 Study Design

This prospective observational cohort study was conducted at the Royal Brisbane and Women's Hospital (RBWH) between August 2022 and March 2025. In addition, 10 historical benign controls were included in this study from a previous study [25]. Ethical approval was obtained under protocols HREC/2021/QRBW/81884 (prospective cohort) and HREC06/QRBW138 (historical controls). All participants provided written informed consent. The study adhered to the Declaration of Helsinki and incorporated TRIPOD reporting principles relevant to exploratory diagnostic prediction modeling.

### 5.2 Participants

#### 5.2.1 Inclusion Criteria

Adults referred to the RBWH Thoracic Medicine Clinic with pulmonary nodules measuring 8–30 mm in diameter were recruited for the study. Eligible patients who agreed to participate and provided written informed consent were consecutively enrolled during the recruitment period. Although this is not a screening cohort, the inclusion criteria mirrored those used in screening programs for solitary or dominant indeterminate nodules undergoing diagnostic work‐up. At baseline, malignancy risk was recorded using the Brock model and clinical protocol prediction (PLCOm2012), where available [49]. The reference standard for malignancy was histopathological confirmation (bronchoscopy with EBUS/robotic guidance or CT‐guided biopsy). Benign status required radiological stability or resolution at 12 months when a biopsy was not obtained. Breath samples were collected before the reference‐standard assessment, and no study‐related intervention occurred between breath collection and reference‐standard determination.

#### 5.2.2 Cohort Accounting for Breath Analyses

Two breath analysis arms were conducted in the same cohort: one using eNose sensors and the other using GC‐MS, with breath collected before biopsy and modality‐specific quality‐control criteria applied as detailed in Section 2.

### 5.3 Breath Collection

The participants fasted for ≥ 6 h, avoided smoking for ≥ 12 h, and abstained from alcohol for > 24 h before breath testing. Collections were performed in the same lung function laboratory and analyzed with a Cyranose 320 following pre‐biopsy procedures [50]. A VOC A2 filter (North Safety, NL) was fitted to the inspiratory port of a Hans Rudolph two‐way nonrebreathing valve (Hans Rudolph Inc., KS, USA). Exhaled breath was routed through PTFE tubing into a 0.5 L Tedlar bag (SKC Inc., PA, USA). A second Tedlar bag was filled with room air under identical conditions to assess background VOCs in the environment.

### 5.4 ENose Analysis

#### 5.4.1 Device Setup and Acquisition

The Cyranose 320 was used to analyze exhaled breath samples. This portable device contains 32 carbon black polymer composite chemiresistors that adsorb exhaled VOCs to varying degrees, producing a characteristic differential electrical pattern. Each participant provided a single full‐breath exhalation that was processed twice; only the second pass was retained for analysis, with the first used to condition the sensors (swelling/relaxation) and stabilize responses across the array. Device operation followed the Amsterdam protocol with local adaptations [50].

#### 5.4.2 Pre‐Processing and Feature Engineering

The raw sensor outputs were exported to JMP Pro, log‐transformed, and then within‐subject mean‐centered to reduce inter‐individual baseline offsets before modeling.

#### 5.4.3 Model Families and Tuning

Three logistic regression models were constructed: (i) clinical‐only, (ii) eNose sensor‐only, and (iii) combined clinical + eNose sensor. The clinical covariates included age, sex, smoking history, and ECOG status. The Brock score and nodule diameter were omitted because they were missing in historical controls. Sensor selection utilized adaptive LASSO with parameter tuning via LOOCV. Model discrimination was evaluated using ROC curves and corresponding AUCs, with 95% CIs, MCRs, and AUC comparisons among the three models. We used adaptive LASSO logistic regression to reduce overfitting and obtain an interpretable subset of predictors.

### 5.5 GC‐MS Acquisition and Processing

#### 5.5.1 Acquisition, Instrumentation, and Identification

VOCs in the breath samples were analyzed using GC‐MS following the principles of the EPA Method TO‐15 [51]. The samples were preconcentrated and cryofocused using a Markes UNITY–CIA Advantage XR system and analyzed on an Agilent 7890–5977A GC‐MS. The data were processed using Agilent MassHunter software. Targeted quantification was performed with a TO‐15 75‐component gas standard (Linde Group). Untargeted discovery applied chromatogram deconvolution with tentative identification against the NIST library and semi‐quantification relative to the internal standard 1,4‐difluorobenzene. All analyses were performed at the Organic Chemistry Laboratory, Public and Environmental Health Reference Laboratories (PEHRL), Queensland Health, Brisbane, Australia.

#### 5.5.2 Feature Curation and Missingness Rule

Following the integration of data from both the malignant and benign cohorts, 296 distinct VOCs were identified. Compounds detected in fewer than 30% of the samples were eliminated, leaving 48 features for hypothesis testing. Given the semi‐quantitative nature of the assay, the features were dichotomized as present (intensity > 0) versus absent for all downstream analyses.

#### 5.5.3 Model Typing and Fine‐Tuning

Three logistic‐regression models were constructed for the GC‐MS data: (i) clinical‐only, (ii) VOC‐only (GC‐MS‐derived VOC features), and (iii) combined clinical + VOC. The GC‐MS clinical covariate set included age, sex, smoking history, nodule diameter, ECOG performance status, Brock score, respiratory comorbidity (Resp CM), and overall comorbidity score (CM score).

### 5.6 Statistical Analysis

#### 5.6.1 Data Pre‐Processing and Model Tuning

For eNose data, sensor outputs were log‐transformed and mean‐centered to mitigate baseline drift. For GC‐MS data, the curated features (see Section 5.5.2) were analyzed as binary presence/absence.

We used clinical‐only, biomarker‐only (eNose sensor‐only for eNose; VOC‐only for GC‐MS), and combined clinical + biomarker models. For the eNose analyses, the clinical covariates used were age, sex, smoking history, and ECOG status. For the GC‐MS analyses, the clinical covariates used were age, sex, smoking history, nodule diameter, ECOG status, Brock score, Resp CM, and overall CM score. For both modalities, models were fitted using adaptive LASSO logistic regression, with inner‐loop LOOCV for tuning and outer‐loop LOOCV to assess overfitting. Bootstrap validation (1000 resamples) was used to estimate optimism via Efron's 0.632+ method [52]. Significance was based on nominal *p*‐values (*p* < 0.05). Model discrimination was assessed using ROC curves and the area under the ROC curve (AUC). Additional performance measures, including sensitivity, specificity, and MCR, were reported where applicable. Predictor stability was defined as inclusion in ≥ 30% of bootstrap refits (1000 adaptive LASSO models). As this was an exploratory proof‐of‐concept study, no formal a priori sample‐size calculation was performed. The sample size was determined by the number of eligible participants recruited during the study period.

#### 5.6.2 Decision‐Threshold Analysis

For GC‐MS classifiers, the optimal probability cut‐point was identified by maximizing Youden's J. The sensitivity, specificity, PPV, NPV, and accuracy were calculated with 95% CIs. Threshold performance was visualized across the probability continuum.

#### 5.6.3 Software and Reproducibility

Analyses were conducted in JMP Pro (version 19.0, SAS Institute, Cary, NC, USA).

## Author Contributions

**Waqar Ahmed Afridi**: methodology, data curation, investigation, formal analysis, visualization, and writing – original draft. **Gunter Hartel**: formal analysis, software, and writing – review and editing. **Xi Zhang**: supervision, data curation, and writing – review and editing. **David Pass**: resources, writing – review and editing. **David Fielding**: resources, supervision, writing – review and editing, validation, conceptualization. **Chamindie Punyadeera**: Supervision, conceptualization, project administration, funding acquisition, writing – review and editing. All authors have read and approved the final manuscript.

## Funding

This research was supported by a Tour de Cure grant (RSP‐130‐2024). Chamindie Punyadeera is currently receiving funding from the National Health and Medical Research Council (APP 2002576 and APP 2012560), the Australian Research Council (IH240100013 and DP250101156), the Garnett Passe and Rodney Williams Foundation, Gallipoli Medical Research Foundation, and the Royal Brisbane Women's Hospital Foundation.

## Ethics Statement

This study was approved by the Human Research Ethics Committee of the Royal Brisbane and Women's Hospital (HREC/2021/QRBW/81884) and the Griffith University Human Research Ethics Committee (HREC/2026/0404). All procedures were conducted in accordance with the relevant guidelines and regulations.

## Consent

All participants provided written informed consent prior to sample collection and the use of de‐identified data and specimens.

## Conflicts of Interest

The authors declare no conflicts of interest.

## Supporting information




**Supporting File 1**: mco271012‐sup‐0001‐SuppMat.docx

## Data Availability

The datasets generated and/or analyzed during the current study are available from the corresponding author upon reasonable request.
