# Supplementary material for: Complementary Breath Profiling Using eNose and Gas Chromatography‐Mass Spectrometry for Noninvasive Triage of Indeterminate Pulmonary Nodules
Source: MedComm (2020). 2026 Sep 23;7(10):e71012. doi: 10.1002/mco2.71012 (PMC13598554; doi:10.1002/mco2.71012)
Supplement: Supplementary file 1 [file MCO2-7-e71012-s001.docx]

**Supplementary Information**

**Complementary Breath Profiling Using eNose and Gas Chromatography-Mass Spectrometry for Non-Invasive Triage of Indeterminate Pulmonary Nodules**

Waqar Ahmed Afridi^1,2^, Gunter Hartel^1,3^, Xi Zhang^1^, David Pass^4^, David Fielding^5^, Chamindie Punyadeera^1*^

Affiliation(s)

^1^ Saliva and Liquid Biopsy Translational Laboratory, Institute for Biomedicine and Glycomics (IBG), Griffith University, Brisbane 4111, Australia

^2^ Virtual University of Pakistan, Islamabad 44000, Pakistan

^3^ QIMR Berghofer Medical Research Institute, Brisbane, 4006, Australia.

^4^ Public and Environmental Health Reference Laboratories (PEHRL), Brisbane, 4108, Australia

^5^ The Royal Brisbane and Women's Hospital, Herston, Brisbane, Queensland 4006, Australia

*Correspondence

Professor Chamindie Punyadeera

Address: Saliva and Liquid Biopsy Translational Laboratory, Institute for Biomedicine and Glycomics (IBG), 46 Don Young Road, Nathan, QLD 4111.

Email: c.punyadeera@griffith.edu.au

**S1. eNose Supplementary Analyses**

**S1.1 Supplementary Tables (eNose)**

**Table S1. AUC comparisons between eNose models**

| Comparison | ΔAUC | χ² | p-value |
| --- | --- | --- | --- |
| Combined (clinical + eNose sensor) vs clinical-only | +0.208 | 9.94 | 0.0019 |
| Combined (clinical + eNose sensor) vs eNose sensor-only | +0.021 | 1.99 | 0.1582 |
| Clinical-only vs eNose sensor-only | –0.187 | 6.84 | 0.0089 |

ΔAUC denotes the difference in AUC between the compared models. χ² and p-values from ROC curve comparisons.

**Table S2. Stability of eNose predictors**

**Inclusion proportions (%) of eNose sensor features across 1,000 adaptive LASSO bootstrap resamples, with predictors retained at ≥30% flagged as stable**.

| Predictor | Selection proportion (%) |
| --- | --- |
| N log S8 | 73 |
| N log S27 | 73 |
| N log S13 | 73 |
| ECOG | 63 |
| N log S6 | 55 |
| Smoking history | 39 |
| Sex | 39 |
| N log S20 | 39 |
| N log S22 | 36 |
| Age | 31 |
| N log S11 | 31 |

Selection proportion (%) = frequency of selection across bootstrap samples. “N log S#” denotes normalised log-transformed eNose sensor outputs used in modelling.

**S1.2 Supplementary Figures (eNose)**

Figure S1. Flow diagram illustrating participant inclusion and exclusion for the eNose and GC-MS analyses. Of the 98 prospectively enrolled participants, 79 were eligible for the eNose analysis after the exclusion of 14 participants because of procedural or eligibility issues, four without breath samples, and one without a valid eNose recording. Ten historical benign controls were subsequently added, yielding a final eNose cohort of 89 participants (55 malignant and 34 benign). For the GC-MS analysis, 75 of the 98 prospectively enrolled participants were included after the exclusion of 14 participants because of procedural or eligibility issues, four without breath samples, and five without valid GC-MS data, resulting in a final cohort of 52 malignant and 23 benign participants.

Figure S2. ROC curve for the clinical-only eNose model, incorporating age, sex, smoking history, and ECOG performance status. The model demonstrated modest discrimination with an AUC of 0.67 (95% CI 0.547–0.773).

Figure S3. ROC curve for the eNose sensor-only model, demonstrating strong discrimination (AUC 0.86; 95% CI 0.755–0.920).

Figure S4. ROC curve from outer-loop leave-one-out cross-validation (LOOCV) for the final eNose model. The validated AUC was 0.67 (95% CI 0.561–0.784), indicating moderate discrimination under internal validation.

**S2. GC-MS Supplementary Analyses**

**S2.1 GC-MS Supplementary Tables**

**Table S3. AUC comparisons between GC-MS models.**

| Model comparison | ΔAUC | χ² | p-value |
| --- | --- | --- | --- |
| VOC-only vs clinical-only | +0.243 | 14.276 | 0.002 |
| Combined (clinical + VOC) vs clinical-only | +0.233 | 14.886 | 0.001 |
| VOC-only vs Combined (clinical + VOC) | +0.010 | 0.815 | 0.3666 |

**Table S4.** Biological interpretation of GC-MS VOCs.

| **Formula** | **Likely chemical class** | **Biological/interpretive note** |
| --- | --- | --- |
| C₆H₁₀O | Carbonyl | Potential lipid-peroxidation or oxidative-stress marker; plausibly tumour-related redox imbalance. |
| C₅H₆O | Oxygenated heterocycle | Oxidative-stress/lipid-peroxidation footprint; reported in lung cancer breath studies. |
| C₅H₁₂ | Alkane | Hydrocarbon feature potentially linked to lipid peroxidation and membrane-damage signatures. |
| C₆H₁₄ | Alkane | Hydrocarbon feature potentially linked to redox stress and lipid-derived processes. |
| C₆H₁₂ | Alkene | Peroxidation/fragmentation product; membrane-lipid turnover. |
| C₇H₁₄ | Alkene/cycloalkane isomer | Hydrocarbon feature potentially related to lipid peroxidation or membrane-derived VOC patterns. |
| C₂Cl₄ | Chlorinated solvent (xenobiotic) | Exposure-related marker; may contribute complementary discriminatory information. |
| C₂HCl₃ | Chlorinated solvent (xenobiotic) | Possible environmental/occupational exposure-related signal. |
| CS₂ | Sulphur-containing compound (xenobiotic) | Exposure/biotransformation indicator; may modulate VOC profiles. |
| C₈H₁₀ | Aromatic hydrocarbon | Possible exposure-related aromatic VOC or confounding covariate. |
| C₈H₁₈ | Alkane | Hydrocarbon feature potentially linked to lipid-derived or exposure-related VOC patterns. |

**S2.2 GC-MS Supplementary Figures**

Figure S5. ROC curve for the GC-MS clinical-only model, demonstrating modest discrimination (AUC 0.73; 95% CI 0.59–0.84).
